# Effects of tobacco smoking on cardiovascular disease in patients with systemic lupus erythematosus: A systematic review and meta-analysis

**DOI:** 10.3389/fimmu.2022.967506

**Published:** 2022-07-27

**Authors:** Wan-tong Zhang, Zhao Liu, Bao-chen Zhu, Zi-yang Cui, Cheng Huang, Xu-jie Wang, Fang Lu, Qiu-yan Li, Wei-liang Weng, Guo-dong Hua, Chun-miao Xue

**Affiliations:** ^1^ Institute of Clinical Pharmacology, Xiyuan Hospital, China Academy of Chinese Medical Sciences, Beijing, China; ^2^ National Medical Products Administration (NMPA) Key Laboratory for Clinical Research and Evaluation of Traditional Chinese Medicine, Beijing, China; ^3^ National Clinical Research Center for Chinese Medicine Cardiology, Xiyuan Hospital, China Academy of Chinese Medical Sciences, Beijing, China; ^4^ Department of Tobacco Control and Prevention of Respiratory Disease, China-Japan Friendship Hospital, Beijing, China; ^5^ Department of Pharmacy, Dongzhimen Hospital, Beijing University of Chinese Medicine, Beijing, China; ^6^ Department of Geriatric Medicine, Beijing Shijitan Hospital, Capital Medical University, Beijing, China; ^7^ Department of Orthopaedics, China-Japan Friendship Hospital, Beijing, China

**Keywords:** systemic lupus erythematosus, tobacco smoking, cardiovascular disease, systematic review, meta-analysis

## Abstract

**Background:**

Patients with systemic lupus erythematosus (SLE) are at increased risk of cardiovascular disease (CVD) compared to the general population. However, little is known about the effects of tobacco smoking on CVD in patients with SLE.

**Objective:**

To systematically review and summarize the available literature regarding the effects of tobacco smoking on developing CVD in patients with SLE.

**Methods:**

We retrieved relevant studies from the following databases: PubMed, EMBASE, Web of Science and China National Knowledge Internet (CNKI) database. Two reviewers independently reviewed the eligible studies, assessed their validity, and extracted relevant data. Sensitivity and subgroup analyses were performed to distinguish sources of heterogeneity.

**Results:**

A total of 10 studies, which comprised 6984 participants, were included in the analysis. The overall quality of evidence was rated as moderate to low. The smoking prevalence among CVD patients was 39.28% (271/690), which was higher than 31.36% (1974/6294) among non-CVD patients. Compared with never-smokers, the risk of developing CVD in current smokers was 1.42 (95% CI: 1.21–1.66). No significant publication bias was found in our meta-analysis.

**Conclusions:**

In spite of the several negative results, this study found that current smokers with SLE have an increased risk of developing CVD, although most of the included studies were in low-to-moderate quality.

**Systematic Review Registration:**

https://www.crd.york.ac.uk/prospero/, identifier CRD42022338109.

## Introduction

Systemic lupus erythematosus (SLE) is a complex chronic autoimmune disease with variable presentations, course and prognosis. Worldwide, 20–150 out of every 100,000 people have SLE (1). Patient with SLE can suffer from clinical manifestations of multiple organs with substantial heterogeneity including arthritis, serositis, nephritis, gastrointestinal and neuropsychiatric problems (2).

A number of previous studies, both case-control and cohort designs, have indicated that SLE is associated with significantly greater CVD risk (3–5). Although the proportion of CVD events in SLE varies, an early study found that around 7% of SLE patients had a cardiovascular event (6). A more recent study from the UK Biobank demonstrated that SLE was associated with an almost 3-fold greater risk of CHD, over 4-fold greater risk of stroke, over 5-fold greater risk of venous thromboembolism (VTE), and an over 15-fold greater risk of peripheral arterial disease (PAD) (7). Therefore, the identification of potential risk factors to protect SLE patients from developing CVD needs careful exploration.

Tobacco smoking is one the most prevalent behaviors and a well-known risk factor associated with cardiovascular diseases, chronic obstructive pulmonary disease (COPD), cancers and diabetes (8, 9). However, little is known regarding the effects of tobacco smoking on developing CVD in patients with SLE. Thus, we conducted a systematic review and meta-analysis to investigate the effects of tobacco smoking on developing CVD in patients with SLE.

## Methods and analysis

### Study overview and registration

This systematic review and meta-analysis was conducted between December 2021 to May 2022, and reported following the Preferred Reporting Items for Systematic Reviews and Meta-Analyses Protocols statement guidelines (10). This study was registered on the PROSPERO **(** https://www.crd.york.ac.uk/prospero/) (CRD42022338109).

### Literature search strategy

To identify relevant articles, the literature dated from the inception of the databases to December 2021 was searched in PubMed, EMBASE, Web of Science and China National Knowledge Internet (CNKI) databases, with no language restrictions.

The keywords and terms utilized in our research were: (systemic lupus erythematosus) AND (smoking or tobacco) AND (cardiovascular disease or heart disease or vascular disease or coronary artery disease or myocardial infarction, congestive heart failure or atherosclerosis). In addition, all references cited were reviewed to identify additional studies that were not included in the above-mentioned electronic databases.

### Study selection

The literature was considered eligible based on the following inclusion criteria: (1) case-control studies or cohort studies; (2) the study provided the proportion of tobacco smoking in SLE patients with CVD and without CVD, respectively; (3) the SLE was diagnosed by international standards, such as the International Classification of Diseases (ICD), the American College of Rheumatology (ACR) standard of the American Society of Rheumatology; (4) all the participants were adults over the age of 18.

The literature was excluded based on the following criteria: (1) case reports, editorials, and reviews; (2) studies where the research subject was not human; (3) studies without control cases; (4) insufficient data or unpublished literature.

Two authors (LIU Z and HUANG C) conducted a comprehensive identification of included publications, independently. Disagreement was resolved through discussion or a third author (XUE CM).

### Data extraction and quality assessment

We extracted the following information from the included study. Two authors (ZHANG WT, ZHU BC) independently extracted data using the standardized covidence data extraction form. A third review author provided a review of the quality assessment and a consensus check.

◼ Funding source
◼ Authors’ declarations of interest
◼ Country, authors and year of publication
◼ Research location
◼ Study design
◼ Inclusion criteria and exclusion criteria
◼ Number of participants
◼ Age and other relevant characteristics of study participants
◼ Proportion of current smokers in SLE patients with CVD
◼ Proportion of current smokers in SLE patients without CVD

The methodological quality of the included studies was assessed using the Newcastle-Ottawa Scale (NOS) (11), which was widely used for assessing the quality of observational studies in a meta-analysis, and was based on selection (4 items), comparability (1 item), and outcome (3 items).25 The star rating system of NOS ranged from 0–9, and a study with 7 stars is regarded as high quality.

### Evaluation of heterogeneity and publication bias

To examine potential sources of heterogeneity observed in the meta-analysis, subgroup analysis was performed based on study region, SLE criteria, different study quality, and proportion of female in total study population. Begg test and Egger test were used to detect publication bias.

### Statistical analysis

In this meta-analysis, Stata SE 16.0 software (StataCorp LP, USA) was used for statistical analysis. The results were reported as relative risk (RR) and 95% confidence interval (CI). The heterogeneity of the results from individual studies was analyzed by I-square statistics, and fixed effect model or random effect model was selected. When I^2^ > 50%, indicating there was heterogeneity, a random effect model was used to improve stability. Otherwise, a fixed effect model was used. Moreover, subgroup analyses were performed to evaluate the robustness of this meta-analysis. Begg test and Egger test were conducted to evaluate publication bias. All P values were calculated by double-tail.

## Results

### Database search results

The initial search was completed on June 1, 2021. We have identified 279 potentially relevant publications. Endnote software was used to eliminate duplicate publications, resulting in 242 records for review.

**Figure 1:**
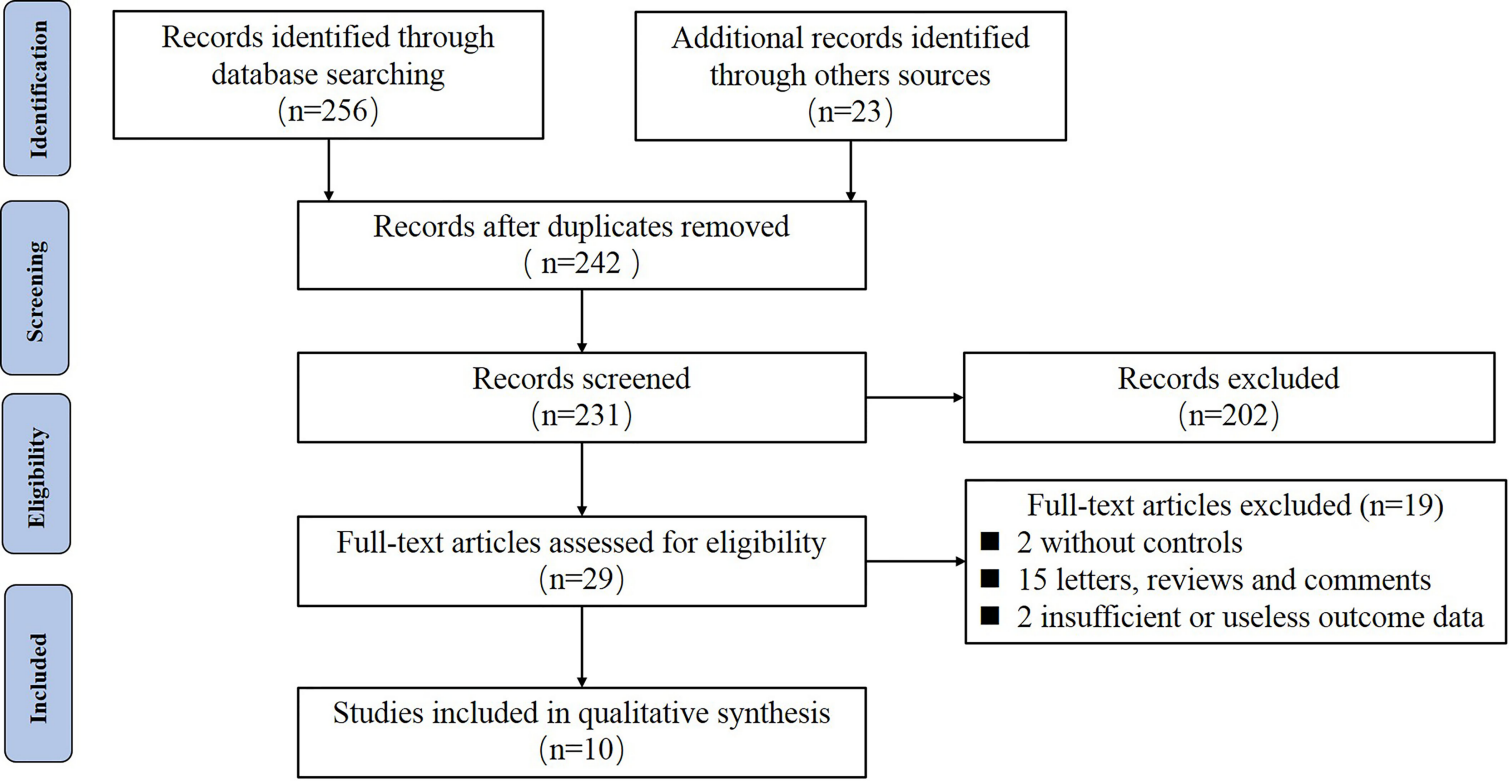
The flow diagram of literature retrieval, screening and exclusion.

After excluding publications that did not meet the inclusion or meet the exclusion criteria, we included 10 studies (12–21) for systematic review and meta-Analysis. A flow diagram illustrating the exclusion of articles with specific reasons was shown in **Figure 1**.

### Study characteristics

The included 10 studies were all cross-sectional studies published between 2004 and 2016. The sample size varied from 57 to 3288, with a total of 6984 patients with SLE. In addition, 4 studies were conducted in Europe, and 6 in North America.

The methods for SLE assessment were consistent, including 6 studies with 1982 ACR criteria (22), and 4 studies with 1997 ACR criteria (23).

As planned, we used Newcastle-Ottawa Quality Assessment Scale (NOS) to evaluate the quality of the cross-sectional studies. Three studies (30%) were considered high quality and 7 studies (70%) were determined as low-to-moderate quality. The main characteristics of the 10 included studies are shown in **Table 1**.

**Table 1:** The main characteristic of all included publications in this study.

First author	Year	Study population	Ethnicity	Region	Mean age	Female%	SLE criteria	NOS score			Total sample size
								Selection	Comparability	Outcome	
Selzer F (12)	2004	Women	White (90.7%)	US	45.2 ± 10.5	100	1982 ACR criteria	**✵✵**	**✵**	**✵✵**	214
Toloza SM (13)	2004	General population	Hispanic, African-American and Caucasian	US	36.5 ± 12.3	89.6	1982 ACR criteria	**✵✵**	**✵**	**✵✵**	546
Szalai AJ (14)	2005	General population	Hispanic, African-American and Caucasian	US	Not reported	43.86	1982 ACR criteria	**✵**	**✵**	**✵✵**	57
Bertoli AM (15)	2009	General population	Texan Hispanics, Puerto Rican Hispanics, African Americans and Caucasians	US	35.7 ± 12.3	90.4	1997 ACR criteria	**✵✵**	**✵✵**	**✵✵✵**	1314
Gustafsson J (16)	2009	General population	European Caucasians Asian	Sweden	45 (31-53)	90	1982 ACR criteria	**✵✵✵**	**✵**	**✵✵✵**	182
Maureen McMahon (17)	2009	Women	Caucasian, Asian, Pacific Islander, African American, Hispanic, Mixed and Other	US	Not reported	100	1997 ACR criteria	**✵✵**	**✵**	**✵✵**	276
Urowitz MB (18)	2010	General population	Caucasian, Black, Hispanic, Asian and other	Canada	34.3 ± 13.3	89.4	1982 ACR criteria	**✵✵✵**	**✵**	**✵**	637
Fernández-Nebro (19)	2015	General population	Caucasian, Amerindian Caucasians, Black Africans, Asians, and others	Spanish	45.0 (35.7–56.5)	90.3	1997 ACR criteria	**✵✵**	**✵**	**✵✵**	3288
Gustafsson JT (20)	2015	General population	European Caucasians (90%)	Sweden	Not reported	86	1982 ACR criteria	**✵✵**	**✵**	**✵✵**	367
Kay SD (21)	2016	General population	White (98%)	Denmark	49.2 ± 14.1	89	1997 ACR criteria	**✵✵✵**	**✵✵**	**✵✵**	103

ACR, American College of Rheumatology. According to NOS, a study can be awarded a maximum of one star for each numbered item within the Selection and Exposure categories. A maximum of two stars can be given for Comparability.

### Tobacco smoking and the risk of developing CVD

**Figure 2:**
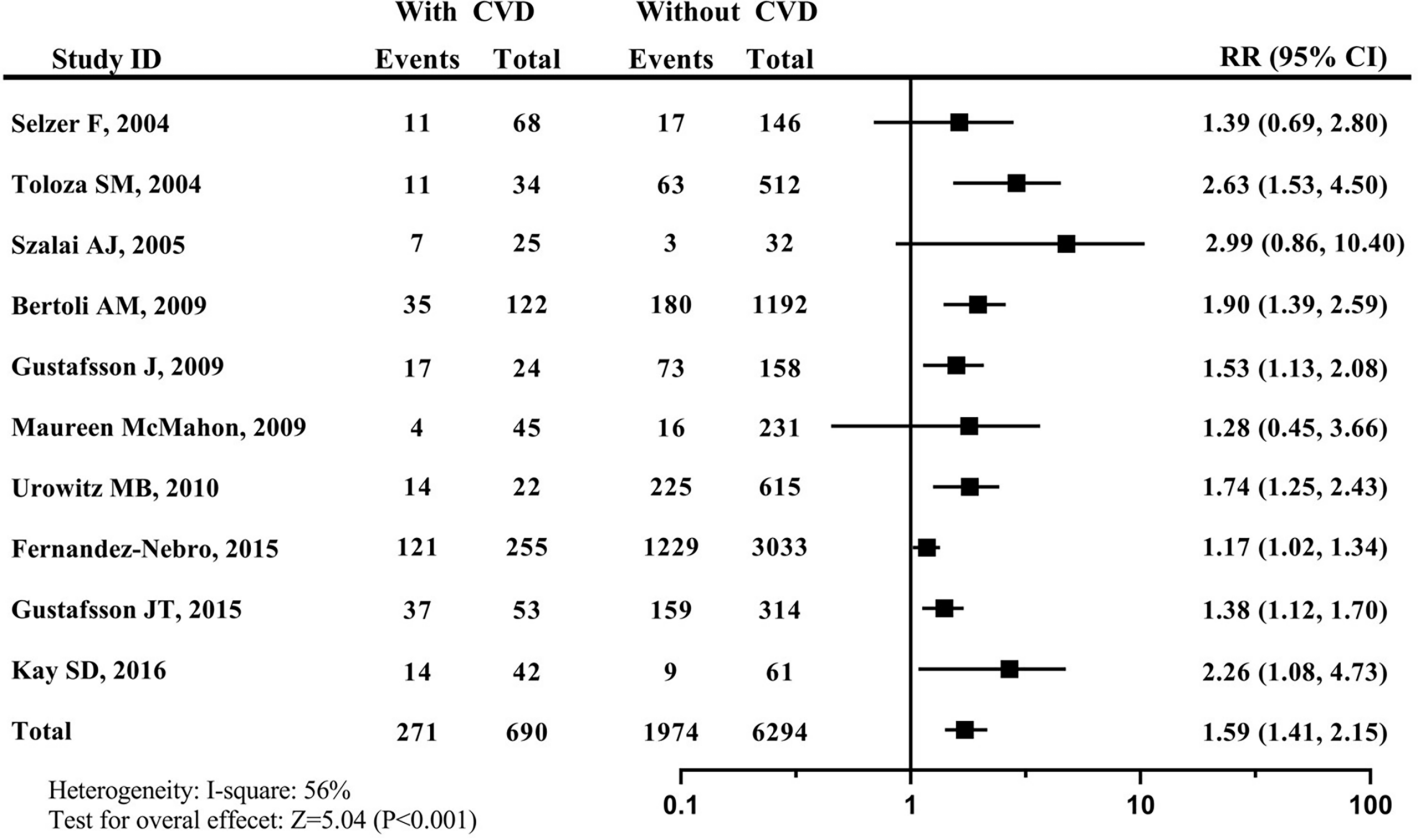
Forest plot for effects of tobacco smoking on developing CVD in patients with SLE. systemic lupus erythematosus, SLE; cardiovascular disease, CVD.

The smoking prevalence among CVD patients was 39.28% (271/690), which was higher than 31.36% (1974/6294) among non-CVD patients. Since the heterogeneity was found among these studies (I^2 =^ 56%), the random effect model was applied for pooled analysis in this study. As a result, compared with never-smokers, the risk of developing CVD in current smokers was 1.59 (95% CI: 1.41–2.15). The forest plot for the relationship between tobacco smoking and developing CVD risk was shown in **Figure 2**.

### Subgroup analysis

**Figure 3:**
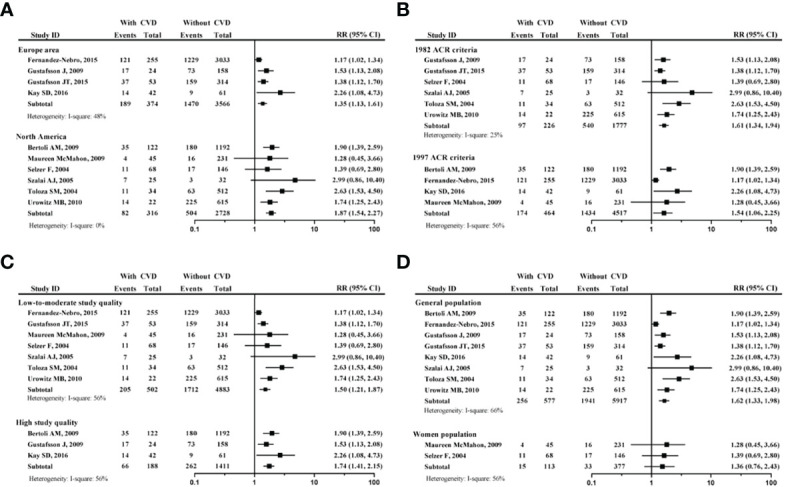
Subgroup analysis of tobacco smoking on developing CVD in patients with SLE. **(A)** forest plot according to the study area; **(B)** forest plot according to different SLE criteria; **(C)** forest plot according to study quality; **(D)** forest plot according to proportion of female participants. systemic lupus erythematosus, SLE; cardiovascular disease, CVD.

According to the study area, the diagnosis criteria of SLE, proportion of female, and quality of study, a subgroup analysis of 10 studies was conducted (**Figure 3**).

The **Figure 3A**, a comparison between the European area and North America area, showed that no matter where the region was, the smoking SLE patients had increased risk of developing CVD (for European area, RR=1.35, 95% CI: 1.13-1.61); for North America area, RR=1.87, 95% CI: 1.54-2.27). The **Figure 3B**, which was a comparison between 1982 ACR criteria and 1997 ACR criteria for SLE, showed that no matter what the diagnosis criteria was, the smoking SLE patients had increased risk of developing CVD (for 1982 ACR criteria, RR=1.61, 95% CI: 1.34-1.94); for 1997 ACR criteria, RR=1.54, 95% CI: 1.06-2.25).

The **Figure 3C**, a comparison between low-to-moderate study quality and high study quality, showed that no matter what the study quality, the smoking SLE patients had increased risk of developing CVD (for low-to-moderate study quality, RR=1.50, 95% CI: 1.21-1.87); for high study quality, RR=1.74, 95% CI: 1.416–2.15). The **Figure 3D**, a comparison between the all-female population and non-100% female population, showed that all-female population had no increased risk of developing CVD (RR=1.36, 95% CI: 0.76-2.43). It should be noted that only 15 smokers were included into this subgroup analysis.

### Publication bias

The Egger linear regression test and Begg funnel chart were adopted to verify whether there was a publication bias in our meta-analysis. The result of Egger linear regression indicated that there was no statistically significant publication bias (P=0.531). The Begg test results also confirmed that there was no statistically significant publication bias (P=0.327). As such, no significant publication bias was found in our meta-analysis.

## Discussion

To the best of our knowledge, this was the first systematic review and Meta-analysis to investigate the effects of tobacco smoking on risk of developing CVD in patients with SLE. Our findings indicated that current smokers with SLE have an increased risk of developing CVD, although most of the included studies were in low-to-moderate quality.

Tobacco smoking is an established risk factor of developing SLE. A large cohort study involving more than 238,000 women in the prospective Nurses’ Health Study indicated that current smokers had increased risk of developing SLE (24). A meta-analysis of seven case-control and two cohort studies found a significant risk for the development of SLE among current smokers compared to non-smokers (OR 1.50, 95% CI 1.09–2.08) (25). A later meta-analysis with newer studies confirmed this finding (26).

In the general population, nearly half of the premature mortality associated with smoking is due to CVD (27). Also, increased risk of premature CVD is well recognized in SLE (28). Therefore, it is important to summarize the effect of smoking on the risk of cardiovascular disease in patients with SLE. Based on the previous evidence, our study further showed that smoking could lead to greater risk of CVD in SLE. Although not surprising, there are several implications and considerations related to our findings. First, the detrimental effects of smoking on SLE should never be underestimated, and we believe that smoking cessation should be one of the primary concerns for physicians treating SLE, and must be a cornerstone of treatment. Second, the quality of the included studies was poor to moderate, particularly most of them had limitations on small sample size and non-representativeness study population, which could decrease reliability of the evidence and restrict the interpretation of these results. Third, with our preliminary data, we call for joint efforts to further explore the association between tobacco smoking and SLE, such as dose–effect relationship between higher smoking packyears and SLE, the detrimental effects of smoking on SLE treatment response, etc.

There are some limitations in this meta-analysis. First, as we mentioned above, the sample size of some included studies was relatively small. Second, due to the lack of relevant studies, secondhand smoking exposure were not explored. Third, the case-control studies included in our meta-analysis may inevitably suffer from several biases by their retrospective design. Finally, as a social phenomenon, tobacco smoking is linked to many confounding factors such as socioeconomic level, stress, bad health habits or lack of therapeutic adherence, which might compromise the findings of our study.

## Conclusion

In spite of the several negative results, this study found that current smokers with SLE have an increased risk of developing CVD, although most of the included studies were in low-to-moderate quality. As such, smoking cessation should be one of the primary concerns for physicians treating SLE, and must be a cornerstone of treatment.

## Data availability statement

The raw data supporting the conclusions of this article will be made available by the authors, without undue reservation.

## Author contributions

W-TZ, ZL, and B-CZ were involved in the methodological design of the systematic review, and conducted the acquisition of data, analyses, and interpretation. W-LW and G-DH directed and organized the systematic review and the methodologist team. FL and C-MX conducted data interpretation, and provided substantial feedback on the drafted manuscript. ZL and B-CZ wrote the manuscript and Q-YL revised the manuscript. All authors were involved in the planning of the study, literature review, interpretation of the findings, manuscript preparation and approved the final version before submission.

## Funding

This study was supported by National Natural Science Foundation of China (82004352), Academic Inheritance Studio of Professor W-LW and China Fundamental Research Funds for Central Public Welfare Research Institutes (ZZ14-YQ-006).

## Conflict of interest

The authors declare that the research was conducted in the absence of any commercial or financial relationships that could be construed as a potential conflict of interest.

The reviewer ZM declared a shared parent affiliation with the author Z-YC to the handling editor at the time of review.

## Publisher’s note

All claims expressed in this article are solely those of the authors and do not necessarily represent those of their affiliated organizations, or those of the publisher, the editors and the reviewers. Any product that may be evaluated in this article, or claim that may be made by its manufacturer, is not guaranteed or endorsed by the publisher.
